# Personal Growth and Wellbeing: An Iterative Mindset Assessment and Perspective

**DOI:** 10.3390/bs15070906

**Published:** 2025-07-04

**Authors:** Kyra Bobinet, Jeni L. Burnette, Whitney Becker, Mallory Rowell

**Affiliations:** 1 Fresh Tri Company, Felton, CA 95018, USA; mrowell@freshtri.com; 2 Department of Psychology, North Carolina State University, Raleigh, NC 27695, USA; jlburne5@ncsu.edu; 3 Independent Consultant, Raleigh, NC 27605, USA

**Keywords:** personal growth, mindsets, wellbeing

## Abstract

Interest in personal growth is expanding in both the popular press and the scientific literature. These expansions incorporate varied theoretical approaches and multiple areas of life. In the current work, we propose a novel perspective that focuses on managing failure to reach self-improvement goals and improving wellbeing. Specifically, we introduce an iterative mindset, which is the belief that making adaptations combined with deliberate practice and neutralizing of failure is critical for lasting transformations. We seek to contribute to the personal growth and mindset literature in two key ways. First, we developed and validated a new measure, called an Iterative Mindset Inventory (IMI), examining factor structure, reliability, and validity. Second, we investigated the links between iterative mindsets, self-improvement, and wellbeing, extending existing work on the power of beliefs to shape self-development. In both studies (Study 1, N = 871; Study 2, N = 345), we incorporated online samples that resembled the adult population of the United States. In Study 1, we found evidence for the proposed theoretical three-factor structure of an iterative mindset, which we label iterate, practice, and assess. In Study 2, using a longitudinal approach across three weeks, we confirmed the three-factor structure and found high test–retest reliability. Iterative mindsets were also positively linked to weight-loss success across both studies and to self-efficacy and wellbeing in Study 2.

## 1. Introduction

As interest in personal growth is expanding in scope and popularity, scientists are asking what personal growth is and how we can facilitate it. Although definitions and conceptualizations vary across disciplines and theoretical perspectives (e.g., Ganti et al., 2025), we suggest that a common theme that emerges is an emphasis on overcoming challenges to improve the self and to flourish. Building on this, we offer a new perspective for examining personal growth, namely an iterative mindset, which is defined as the belief that lasting transformations depend on continually adapting, engaging in intentional practice, and responding to failures with neutrality and learning rather than discouragement.

Using an iterative mindset approach, we build on existing perspectives of personal growth in three key ways. First, whereas some earlier viewpoints emphasize end states such as self-actualization (e.g., Maslow, 1961), we underscore the importance of the process and learning through iteration. Indeed, the first fundamental component of an Iterative Mindset is a belief that continuously refining and adjusting one’s strategies is valuable when seeking long-term behavior changes. This try-and-tweak iterative mentality is a process, rather than outcome-oriented, and fundamentally adaptive. Second, theories rooted in positive psychology suggest that personal growth is one of six key components to wellbeing and define it as continued development and seeking improvement (Ryff, 1989). We suggest that ongoing growth requires emotional resilience in the wake of inevitable setbacks, including a self-compassionate approach to failure management or, more specifically, a recognition that failure is a chance to make adjustments. The second component of an Iterative Mindset is failure assessment, which seeks to address the emotional reactions to setbacks, such as shame, and instead foster a belief that setbacks are not permanent. Such judgment-free and emotionally regulated assessments of failure pave the way for iteration. Third, we emphasize that lasting behavior change and enduring self-improvement require habit building (Wood, 2024). This idea can also be seen in the early perspectives of personal growth, such as Aristotle’s emphasis on the need to do good repeatedly for character development. An Iterative Mindset emphasizes the importance of behaviors becoming automatic and more efficient, creating resilience during motivation lapses (e.g., Clear, 2018; Lally et al., 2010; Wood, 2024). Overall, an Iterative Mindset describes emotion-regulation strategies and beliefs around change and improvement. Those with an Iterative Mindset expect to figure out what works but, at the same time, anticipate that challenges will arise that require emotion regulation and iterations on initial strategies in order to succeed.

The primary goal of the current work is to develop an Iterative Mindset measure that seeks to capture the outlined three components, namely, iteration, assessment, and practice. The theoretical foundation of this newly developed measure draws on our integration of neuroscience findings related to the habenula with the longstanding mindset literature. Neuroplasticity goes hand in hand with mindsets, in that both recognize the potential for change and both seek to answer questions about how to facilitate adaptive responses to failure. For example, a pivotal area in our development of the Iterative Mindset draws upon findings on a significant brain area, the habenula, which is activated by perceived disappointment or frustration (Groos & Helmchen, 2024; Weidacker et al., 2021). Once triggered, the habenula shuts off one’s motivation to persist (Bobinet & Greer, 2023; Huang et al., 2019; Webster et al., 2021; Zapata & Lupica, 2021). Similarly, mindsets, defined as beliefs about the changeable (i.e., growth mindset) vs. stable (i.e., fixed mindset) nature of attributes, traits, abilities, emotions, and more (e.g., Kyler & Moscicki, 2024), shape motivation in the wake of setbacks (Dweck, 1999). For example, growth mindsets set up a self-regulatory pattern of setting goals focused on learning rather than outperforming others, striving to reach these goals by focusing on the process and responding to setbacks with persistence, and finally, by continuing to expect and believe in the potential for future improvement (e.g., Burnette et al., 2013). Overall, growth mindsets are important because they are meaning-making systems that influence how individuals approach goals and challenges, with downstream implications for success, although not all work finds positive effects of growth mindsets, especially when considering academic performance (e.g., Sisk et al., 2018).

In the current work, we move beyond beliefs about the changeable or fixed nature of attributes, outlining how mindsets encompass not just mental patterns but also emotional reactions and behavioral components (Dweck, 1999, 2016). We suggest that the Iterative Mindset is unique from growth mindsets, which are beliefs about the potential to change. Iterative Mindsets encompass related beliefs about the importance of learning through iteration but also include the need to assess failure without judgment and to respond with effortful practice to build habits and automaticity. In the current work, we suggest that an Iterative Mindset can help override the habenula’s orientation towards shame and shutdown and instead encourage curiosity, emotion regulation, and the creativity to try new approaches. All three components are critical for sustained motivation, behavioral change, and lasting transformations. For example, participants with stronger iterative mindsets remain optimistic in the face of challenges, noting things like “I’ve overcome it once, I know how to get through it”, and iterative mindset training helped individuals in habit formation (Bobinet & Greer, 2023).

Our first goal is to create and test an Iterative Mindset measure that captures the proposed three components of the definition reviewed above—iteration, assessment, and practice (Aim 1). We suggest that the Iterative Mindset is distinct from existing mindsets of attributes such as intelligence (Dweck, 1999) and the fundamental nature of people (Chiu et al., 1997)—the two most studied mindsets currently in the literature (Molden & Dweck, 2006). We also expect that the Iterative Mindset is unique and distinct from personality traits such as the Big Five (Rammstedt, 2007). Our second goal is to provide construct validity (Aim 2). Our third goal is to illustrate initial links to personal growth-related outcomes. With our emphasis on self-improvement via lasting behavior change, we hone in on weight loss, a goal that requires sustainable strategies and a willingness to try new things when inevitable obstacles arise (Martin et al., 2018). Weight loss, as a personal goal, is one that is rife with setbacks and is often accompanied by shame (Goodrick et al., 1992). Thus, an Iterative Mindset, with its focus on reframing the meaning of failure to reduce shame, in conjunction with iteration and automaticity, should be related to more successful weight-loss journeys. Early exploratory work supports this idea, with Iterative Mindsets being positively linked to successful weight loss (Bobinet & Greer, 2023). However, this early work did not incorporate a validated assessment, and thus, we seek to replicate the link between Iterative Mindsets and weight-loss journeys using the newly developed Iterative Mindset scale and its components (Aim 3).

Finally, we examine links between Iterative Mindsets and wellbeing, which we define as a multidimensional construct consisting of positive feelings and life satisfaction, as well as the absence of burnout and stress (Jarden & Roache, 2023). We use this broader conceptualization of wellbeing, as it is closely aligned with our conceptualization of personal growth. For example, we suggest that personal growth, especially self-improvement, requires a critical lens and self-reflection. However, such processes may reveal inadequacies and require adaptive coping to avoid negative emotions and evaluations. Mindsets can serve as a protective factor, and thus, we expect Iterative Mindsets to be positively linked to wellbeing. Additionally, we explore if this link is driven, in part, via self-efficacy. We hypothesize that iterative thinking, with its affective (i.e., failure assessment) and behavioral (i.e., practice and iteration) components, contributes to more positive expectations and cognitions, such as self-efficacy (i.e., confidence in one’s abilities; Bandura, 1997). Self-efficacy is positively linked to a host of wellbeing outcomes, including life satisfaction (e.g., O’Sullivan, 2011), and is negatively linked to burnout and stress (e.g., Schwarzer & Hallum, 2008; Shoji et al., 2016). Thus, we test an overall model linking Iterative Mindsets to wellbeing via self-efficacy (Aim 4). We tested the first three aims across both studies and Aim 4 in Study 2. Both studies were performed in accordance with the ethical standards as laid down in the 1964 Declaration of Helsinki and its later amendments or comparable ethical standards. Informed consent was obtained from all participants included in the study. An independent ethical review board determined the studies to be exempt.

## 2. Study 1

### 2.1. Overview and Hypotheses

In Study 1, we explore the factor structure of the Iterative Mindset Inventory (IMI), testing our first hypothesis that the Iterative Mindset is composed of three components of iteration, assessment, and practice. To reach this goal, we randomly split the overall sample to conduct an exploratory factor analysis and confirmatory factor analysis (Brown, 2015; Carpenter, 2018; Ford et al., 1986; Goretzko, 2020; Hu & Bentler, 1999; Meichsner et al., 2016; White et al., 2020). Second, we hypothesized that Iterative Mindsets are related to, yet distinct from, other types of mindsets and existing personality assessments (Spinath et al., 2003). Specifically, we focused on the mindsets of intelligence and people, as these are two of the most studied mindsets in the literature (see Molden & Dweck, 2006), and we examined links to the Big Five personality traits, as this is a dominant model in the personality literature (Goldberg, 1990). Third, we expected Iterative Mindsets to predict weight-loss success (Bobinet & Greer, 2023). Finally, we explored the potential demographic correlates of Iterative Mindsets.

### 2.2. Methods Study 1

#### 2.2.1. Participants

We recruited a large online sample, using CloudResearch, that resembled the adult population of the United States according to the most recent population survey conducted by the U.S. Census Bureau. Namely, we recruited on the following demographics: gender, age, ethnicity (Hispanic vs. not), and race (allowing each respondent to select more than one race). To improve and assess online data quality, we utilized multiple best practices (Brühlmann et al., 2020). We excluded participants who provided poor-quality data, including those who (a) attempted the survey more than once, (b) skipped open-ended questions, and (c) failed a check for eligibility. This resulted in 79 individuals being excluded from analyses.

Data screening resulted in a final sample of 871 included participants. The mean age of the sample was 45.49; SD = 15.38 (range 18–82). In terms of gender, 47.9% of participants were men and 52.1% were women. In terms of race/ethnicity, 78.1% of participants identified as White, 14.7% as Black or African American, 0.1% as American Indian or Alaskan Native, 4.1% as Asian, and 3.0% as a race/ethnicity not listed. The majority had a bachelor’s degree or higher (60%) and made USD 50,000 or more in annual income (approximately 62%).

#### 2.2.2. Measures

We measured three types of mindsets: Iterative Mindsets, Mindsets of Intelligence, and Mindsets of People. We also included A Big Five Personality Assessment, weight-loss journeys, and demographics.

Iterative Mindsets. Theoretically, we expected the Iterative Mindset Inventory to consist of three critical ideas: (1) practice, which involves putting strategies into action and repeating them until they become habit, (2) iterate, which focuses on identifying and adapting strategies for successful habit formation, and (3) assess, which emphasizes reframing failures with positive cognitions. To create initial items, we utilized a deductive approach for item generation, drawing on both experts in the field, as well as general mindset theory (e.g., Dweck, 1986, 2016; Molden & Dweck, 2006). Our initial set consisted of a total of 54 items, which exceeds the recommendation of including at least twice as many items in the initial pool as in the expected final measure (Kline, 2013; Schinka et al., 2013). The items were measured on a 7-point Likert scale (1 = strongly disagree, 7 = strongly agree; see Table 1 for items). We provide additional details in the analysis section.

Mindsets of Intelligence. The participants responded to three items from the validated mindsets of the intelligence scale, rated on a 7-point Likert scale (1 = strongly disagree, 7 = strongly agree; Dweck, 1999). An example item is, “You have a certain amount of intelligence, and you can’t really do much to change it.” We recoded with higher scores, indicating a stronger growth mindset (α = 0.97).

Mindsets of People. The participants responded to eight items from the mindset of person scale, rated on a 7-point Likert scale (1 = strongly disagree, 7 = strongly agree; Chiu et al., 1997; Dweck, 1999). An example item is, “No matter what kind of person someone is, they can always change very much.” We recoded with higher scores indicating a stronger growth mindset (α = 0.95).

Big Five Personality. Participants responded to the BFI-10, which is a brief version of the Big Five Personality Inventory (Rammstedt, 2007). The items are assessed on a 5-point Likert scale (1 = strongly disagree, 5 = strongly agree). We recoded the negatively worded item and created five personality assessments. With only two items per personality trait, it is perhaps not surprising that reliabilities were low, ranging from 0.46 for agreeableness to 0.79 for neuroticism.

Weight-Loss Journeys. The participants responded to one question measuring weight-loss success (Bobinet & Greer, 2023). The question asked, “Which best describes your experience with weight loss? Please read all the options and choose just one that best describes you.” The participants could choose from the following answer options: (1) never tried (I’ve never tried to lose weight), (2) strugglers (I’ve struggled to lose weight without success, (3) relapsers (I’ve lost weight before, but gained it back, (4) achievers (I’m actively losing weight now), and (5) succeeders (I’ve lost weight and kept it off for more than 1 year). We recoded these into a two-category variable: strugglers/relapsers (0) and succeeders/achievers (1), with those who never tried to lose weight coded as missing.

Demographics. The participants completed age, gender, race, education level, and income.

### 2.3. Results Study 1

#### 2.3.1. Data Analysis Strategy

We used random sampling in SPSS 29 to break the overall sample into two separate subsamples, conducting an exploratory factor analysis on the first sample and a confirmatory factor analysis on the second sample (Yang, 2005). We had N = 435 participants in the first (exploratory) subsample and N = 436 participants in the second (confirmatory) subsample (Goretzko, 2020). After finalizing the factors, we used the full data set (N = 871) for the remaining analyses.

#### 2.3.2. Aim 1: Factor Structure

We first conducted exploratory factor analyses using maximum likelihood with promax rotations. We chose maximum likelihood because we expected items to be correlated, and this approach fits well with CFA models (e.g., Meichsner et al., 2016; White et al., 2020). We relied on the following a priori criteria for item and factor retention (Carpenter, 2018). Namely, using an a priori threshold, we kept items that clearly loaded onto a single factor (≥0.40; Ford et al., 1986), had no significant cross-loadings, and communalities that were ≥0.40. To keep a factor, we primarily focused on scree plots, rather than eigenvalues, which can be misleading (van der Eijk & Rose, 2015). This method was chosen given that it provides a more conservative and accurate estimate of factors (Ruscio & Roche, 2012; van der Eijk & Rose, 2015). Also, in line with best practices, we only retained a factor if at least three items loaded on it, as fewer than this can result in unstable and difficult to interpret factors (Fabrigar et al., 1999).

Using the initial 54 items, the analysis applying an eigenvalue greater than one rule resulted in eight factors, but analyzing the scree plot suggested a three- or four-factor solution. However, factors 4–8 had fewer than three items that met the inclusion criteria. Next, we ran another exploratory factor analysis with the items that met the inclusion criteria and loaded on these three factors. This resulted in two more items that needed to be deleted—one that had a communality of less than 0.40 and one that was cross-loaded. We dropped these and ran one more EFA. This resulted in a clear three-factor solution with eigenvalues ranging from 1.37 to 11.84, explaining 55.98% of the variance. The items and factor loadings from the pattern matrix from this analysis can be seen in Table 1. Most of the factor loadings were especially high. Thus, in this final EFA, we focused on items with loadings of 0.60 or more (and no cross loadings) and those that had clear theoretical links, as well as less overlap with other items on the same factor (Samuels, 2017). The more stringent factor loading of 0.60, compared to 0.40 in earlier iterations, in conjunction with a reliance on theory and uniqueness, allowed us to create a more parsimonious assessment. No modification indices were used to improve fit.

The first factor, what we called practice, had ten items, with factor loadings ranging from 0.640 to 0.851. The second factor, what we called iterate, had four items ranging from 0.617 to 0.781, and the final factor, which we called assess, had four items with factor loadings ranging from 0.695 to 0.823. To keep the number of items the same across the three factors, we kept the top four items with the highest factor loadings for practice to create a 12-item scale with 4 items per factor. The final factor loadings for practice ranged from 0.779 to 0.806. The first factor (practice) is interpreted as continuous effort around a behavioral practice to automate and eventually make it a habit. The second factor (iterate) is interpreted as experimentation, adjusting, and adapting behaviors in the face of obstacles to find what works and motivate towards staying in effort (i.e., more practice). The third factor (assess) is interpreted as managing difficult emotions by neutralizing perceived failure.

**Figure 1:**
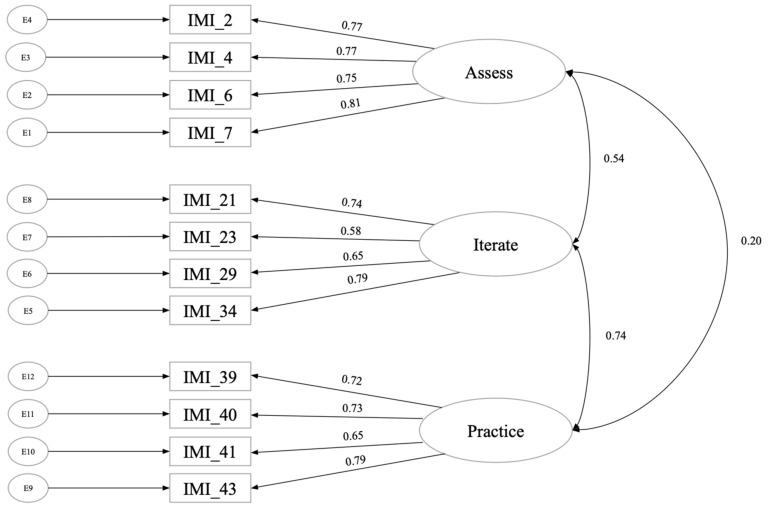
Study 1 CFA results.

We then used the confirmatory subsample to run a CFA, testing whether this three-factor model with 12 items acceptably fit the data (e.g., Hu & Bentler, 1999). We used multiple linear regression (MLR) to estimate the model parameters and goodness of fit, using the following thresholds: RMSEA ≤ 0.06 (90% CI ≤ 0.07), CFI ≥ 0.97, and TLI ≥ 0.96 (Hu & Bentler, 1999; Brown, 2015). The model showed acceptable fit, CFI = 0.97, RMSEA = 0.05, and the factor loadings ranged from 0.578 to 0.815 (see Figure 1).

The reliability for the practice factor was α = 0.81 (ω = 0.81), iterate was α = 0.78 (ω = 0.78), and assess was α = 0.85 (ω = 0.85). Practice, iterate, and assess were all highly correlated with the overall Iterative Mindset Inventory (IMI; Table 2), suggesting that an overall measure could also be appropriate depending on theory and relations with other constructs. Theoretically, we conceptualize an Iterative Mindset as an overall construct that captures each of the underlying ideas (α = 0.83; ω = 0.81). We report correlations with both the overall scale and each factor, as this work is exploratory, and such information can inform theory and intervention development.

#### 2.3.3. Aim 2: Validity

See Table 2 for the means and standard deviations, as well as the correlations among constructs. Overall, the IMI and factors are clearly assessing a new type of mindset—the correlations with mindsets of intelligence and people were small to negligible, ranging from −0.04 to 0.23. In terms of links to personality, the correlations ranged from −0.47 to 0.37, with the strongest links being with neuroticism (See Table 2). These correlations are in expected directions yet offer discriminant validity as “they do not correlate strongly enough to suggest that they measure the same construct” (McKenny et al., 2013, p. 156).

#### 2.3.4. Aim 3: Weight-Loss Journeys

Next, we investigated whether the Iterative Mindset was related to weight-loss journeys. We performed logistic regressions using two categories, comparing those who succeeded (i.e., succeeders/achievers) to those who struggled (i.e., relapsers/strugglers). For the overall IMI, the model is statistically significant χ^2^(1) = 10.60, *p* = 0.001. The model explained 2% (Nagelkerke R squared) of the variance and correctly classified 61% of cases. For every unit increase in IMI, the odds of succeeding or achieving increased by 1.41. For practice, the logistic regression model was not statistically significant χ^2^(1) = 2.01, *p* = 0.157. For iterate and assess, however, the logistic regression models were statistically significant χ^2^(1) = 9.42, *p* = 0.002 and χ^2^(1) = 7.38, *p* = 0.007, respectively. For iterate, the model explained 2% (Nagelkerke R squared) of the variance in weight management segmentation and correctly classified 61% of cases. For every unit increase in iterate scores, the odds of succeeding or achieving weight loss increased by 1.36. For assess, the model explained 2% (Nagelkerke R squared) of the variance in weight management segmentation and correctly classified 60% of cases. For every unit increase in assess scores, the odds of succeeding or achieving weight loss increased by 1.17. These findings highlight the potential utility of understanding each facet, with an emphasis on iteration and assessing failure for understanding weight-loss journeys.

#### 2.3.5. Exploratory Question: Do Demographics Relate to IMI Scores?

We also explored whether Iterative Mindsets was related to demographics. Age was not significantly related to the overall IMI (*r* = 0.03, *p* = 0.264) nor to iterate (*r* = 0.01, *p* = 0.762) or assess (*r* = −0.01, *p* = 0.746), but was significantly and positively related to practice (*r* = 0.10, *p* = 0.005). Next, we ran an independent t-test exploring gender differences. There were no significant differences by gender for the IMI (*t*(869) = 0.21, *p* = 0.831, *d* = 0.014), practice (*t*(869) = −1.00, *p* = 0.320, *d* = −0.067), iterate (*t*(869) = 0.37, *p* = 0.710, *d* = 0.025), or assess (*t*(869) = 0.70, *p* = 0.485, *d* = 0.047). Furthermore, we ran a MANOVA to examine the differences based on race. There were no significant differences in IMI or the factors by race (*p* values for univariate tests ranged from 0.223 to 0.714). However, considering the unequal cells, these results should be interpreted with caution.

### 2.4. Discussion Study 1

Overall, the main goal of Study 1 was to develop an Iterative Mindset Inventory, examining factor structure and construct validity. To reach this goal, we ran an EFA and CFA, with results supporting a 12-item, three-factor scale that we suggest theoretically captures iteration, assessment, and practice. The IMI and factors were distinct from mindsets of intelligence and people but were moderately correlated with the Big Five personality traits, especially neuroticism. Next, we examined links to weight-loss journeys, replicating past work suggesting that Iterative Mindsets can help to distinguish those who are more successful from those who struggle. Finally, the IMI was not consistently or robustly correlated with age, gender, or race.

## 3. Study 2

In Study 2, we again ran a CFA to confirm the factor structure and model fit. Furthermore, we also examined test–retest reliability. Additionally, for discriminant validity, we again assessed neuroticism, as this had one of the highest correlations in Study 1. Extending Study 1, we examined links between an Iterative Mindset and wellbeing. In doing so, we also included constructs previously linked to growth mindsets and those that are theoretically related to an Iterative Mindset and to wellbeing. First, we examined grit, which is defined as “passion and perseverance toward long-term goals” (Duckworth et al., 2007, p. 1087). We expected the overall IMI and each factor to correlate positively with grit. Second, we examined goal orientations, which consist of both learning and performance goals (Dweck, 1986; Dweck & Leggett, 1988; Elliott & Dweck, 1983). Learning goals focus on the process of acquiring a skill, whereas performance goal orientations place an emphasis on eliciting favorable views of one’s competence and abilities and avoiding negative ones (VandeWalle et al., 2001). We expected IMI and the factors to correlate positively with learning goals but not to be strongly related to performance goals. We also anticipated that Iterative Mindsets would again relate to weight-loss journeys. We expected an Iterative Mindset to relate positively to wellbeing and self-efficacy, and we also explored a statistical test of mediation in which self-efficacy is one potential process by which Iterative Mindsets are linked to wellbeing. We separated the predictor (iterative mindsets) and outcomes (self-efficacy and wellbeing) across two time points to avoid common method variance (Kock et al., 2021).

### 3.1. Methods Study 2

#### 3.1.1. Participants

We recruited a sample that resembled the adult population of the United States according to the most recent population survey from the U.S. Census Bureau (Blakeslee et al., 2023; Jones et al., 2021) using CloudResearch, the same online survey platform that was used in Study 1. Namely, we recruited on the following demographics: gender, age, ethnicity (Hispanic vs. not), and race (allowing each respondent to select more than one race). Like Study 1, we reviewed the dataset for data quality and carelessness before running the analyses (Brühlmann et al., 2020). Specifically, we excluded participants who completed or attempted to complete the survey more than once, provided strange/nonsensical open-ended answers, whose self-reported age and gender did not match the age and gender they reported on their CloudResearch profiles, and who completed the survey multiple times. This resulted in 57 participants being excluded. We had 345 participants at Time 1 and 311 participants completing the follow-up survey three weeks later, resulting in an attrition of 34 participants or 11%. The mean age of the sample at Time 1 was 46.04; SD = 15.66 (range 20–80). In terms of gender, 49.6% of participants were men and 50.4% of participants were women. In terms of race/ethnicity, 80.6% of participants identified as White, 12.5% as Black or African American, 0.3% as American Indian or Alaskan Native, 4.3% as Asian, and 2.3% as a race/ethnicity not listed.

#### 3.1.2. Measures

**Time 1 Assessments.**^1^ Below are the measures we collected at Time 1.

Iterative mindsets. Participants responded to the 12-item Iterative Mindset Inventory (IMI) from Study 1. The reliability of the total IMI was α = 0.84 (ω = 0.80). The reliability for practice was α = 0.84 (ω = 0.84), iterate was α = 0.87 (ω = 0.87), and assess was α = 0.86 (ω = 0.86).

Neuroticism. The participants completed an eight-item neuroticism subscale of the Big Five Inventory (John & Srivastava, 1999). The items were measured on a 5-point Likert scale (1 = disagree strongly, 5 = agree strongly). Items were recoded so that a higher score indicates greater neuroticism (α = 0.91).

Grit. The participants completed the Grit Scale (Duckworth & Quinn, 2009). The eight items were measured on a 5-point Likert scale (1 = very much like me, 5 = not like me at all). Items were reverse-coded, so a higher score indicates more grit (α = 0.86).

Goal orientations. The participants completed a measure of learning and performance goal orientation, each consisting of eight items (Button et al., 1996). The items were measured on a 7-point Likert scale (1 = strongly disagree, 7 = strongly agree). Higher scores indicate stronger learning goal orientations (α = 0.91) and performance goal orientations (α = 0.86).

Weight-loss journey. The participants completed the same measure from Study 1.

Demographics. The participants reported age, race, and gender.

**Time 2 Assessments.** Below, we detail the measures collected at the 3-week follow-up.

Iterative mindsets. The overall measure exhibited strong reliability (α = 0.86, ω = 0.80). We also separated these into the three factors of practice (α = 0.84, ω = 0.86), iterate (α = 0.88, ω = 0.87), and assess (α = 0.86, ω = 0.86).

General self-efficacy. The participants completed the 10-item Generalized Self-Efficacy Scale (Schwarzer & Jerusalem, 1995). The items were measured on a 4-point Likert scale (1 = not at all true, 4 = exactly true). The items are summed so a higher score indicates more self-efficacy (α = 0.93).

Wellbeing. The participants completed three separate measures to assess wellbeing: the 14-item short version of the Scales of Well-Being (Longo et al., 2018), the 12-item short version of the Burnout Assessment Tool measuring general burnout (BAT, Schaufeli et al., 2019), and the 4-item version of the Perceived Stress Scale (PSS-4, Cohen et al., 1983). All measures were on a five-point Likert scale, although we did need to recode a 0 to 4 for the PSS-4 to match the 1–5 scale. We then combined all measures to create a mean score of wellbeing, with higher scores indicating greater wellbeing (α = 0.96).

Demographics. The participants completed the same demographic questions from Time 1.

### 3.2. Study 2 Results

#### 3.2.1. Aim 1: Factor Structure and Test–Retest Reliability

**Figure 2:**
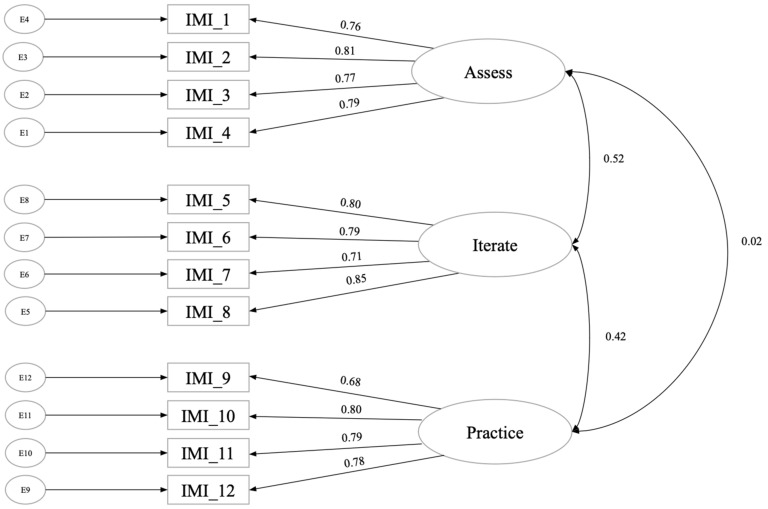
Study 2 CFA results.

For the CFA, we used a multiple linear regression (MLR) to estimate model parameters and goodness of fit, using the following thresholds as a guide: RMSEA ≤ 0.06 (90% CI ≤ 0.07), CFI ≥ 0.97, and TLI ≥ 0.96 (Hu & Bentler, 1999; Brown, 2015). To validate the structure of the scale, we fit a three-factor model. This model illustrated a decent fit based on recommendations (Sun, 2005), CFI = 0.96, RMSEA = 0.07, with the factor loadings ranging from 0.678 to 0.852 (see Figure 2). While our CFI and RMSEA values are slightly outside of the thresholds listed above, which indicate “good model fit,” the RMSEA and CFI values still indicate acceptable fit. The test–retest reliability across the three-week period was *r* = 0.82, *p* < 0.001.

#### 3.2.2. Aim 2: Construct Validity

See Table 3 for correlations. IMI and the factors were negatively linked to neuroticism, with correlations ranging from −0.07 to −0.60. Additionally, as expected, grit was positively related to IMI, (*r* = 0.52, *p* < 0.001), and to the factors of practice, (*r* = 0.13, *p* = 0.017), iterate, (*r* = 0.49, *p* < 0.001), and assess (*r* = 0.46, *p* < 0.001). Learning goal orientation was also positively correlated with IMI (*r* = 0.56, *p* < 0.001) as well as practice (*r* = 0.34, *p* < 0.001), iterate (*r* = 0.65, *p* < 0.001), and assess (*r* = 0.30, *p* < 0.001). However, performance goal orientation was not correlated with the total IMI (*r* = −0.04, *p* = 0.509) nor with iterate (*r* = −0.01, *p* = 0.810). It was, however, correlated positively with practice (*r* = 0.21, *p* < 0.001) and negatively with assess (*r* = −0.18, *p* < 0.001).

#### 3.2.3. Aim 3: Weight-Loss Journeys

We again performed a logistic regression to assess the effects of IMI and its factors. For the overall IMI, the logistic regression model was statistically significant χ^2^(1) = 16.13, *p* < 0.001. The model explained 8% (Nagelkerke R squared) of the variance and correctly classified 61% of cases. For every unit increase in overall IMI scores, the odds of succeeding or achieving increased by 1.87. For practice, the logistic regression model was significant χ^2^(1) = 3.92, *p* = 0.048. The model explained 2% (Nagelkerke R squared) of the variance in weight-loss segmentation and correctly classified 53% of cases. For every unit increase in practice scores, the odds of succeeding or achieving weight loss increased by 1.32. For iterate, the logistic regression model was statistically significant χ^2^(1) = 10.80, *p* = 0.001. The model explained 5% (Nagelkerke R squared) of the variance in weight loss segmentation and correctly classified 58% of the cases. For every unit increase in iterate scores, the odds of succeeding or achieving weight loss increased by 1.57. For assess, the logistic regression model was statistically significant χ^2^(1) = 11.39, *p* < 0.001. The model explained 6% (Nagelkerke R squared) of the variance in weight-loss segmentation and correctly classified 58% of cases. For every unit increase in the assess scores, the odds of succeeding or achieving weight loss increased by 1.33.

#### 3.2.4. Aim 4: Iterative Mindsets, Self-Efficacy, and Wellbeing

IMI was positively correlated with general self-efficacy (*r* = 0.64, *p* < 0.001) and wellbeing (*r* = 0.68, *p* < 0.001). Practice (*r* = 0.23, *p* < 0.001), iterate (*r* = 0.68, *p* < 0.001), and assess (*r* = 0.48, *p* < 0.001) were all positively correlated with general self-efficacy. Practice (*r* = 0.24, *p* < 0.001), iterate (*r* = 0.61, *p* < 0.001), and assess (*r* = 0.58, *p* < 0.001) were also positively correlated with wellbeing.

**Figure 3:**
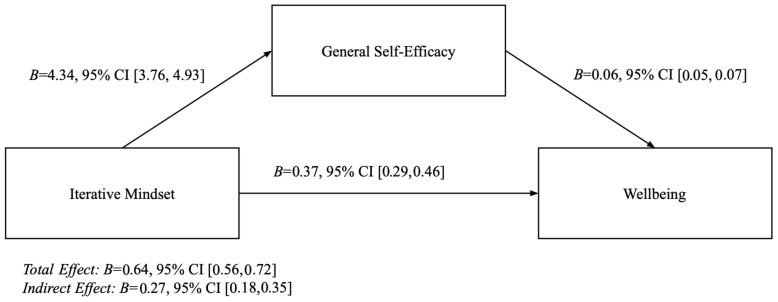
Study 2 mediation model—no control variables.

We used Hayes’ PROCESS Model 4 (Hayes, 2018) to investigate the indirect effects of the overall Iterative Mindset Inventory via self-efficacy on wellbeing. Stronger Iterative Mindsets were related to greater general self-efficacy (*B* = 4.34, *t*(309) = 14.65, *p* < 0.001, 95% CI [3.76, 4.93]). General self-efficacy was related to greater wellbeing (*B* = 0.06, *t*(308) = −9.14, *p* < 0.001, 95% CI [0.05, 0.07]). There was a significant and positive indirect effect of Iterative Mindsets on wellbeing through general self-efficacy (B = 0.27, 95% CI [0.18, 0.35]). There was also a significant positive total effect (*B* = 0.64, *p* < 0.001, 95% CI [0.56, 0.72]) and direct effect (*B* = 0.37, *p* < 0.001, 95% CI [0.29, 0.46]). See Figure 3. The effects hold when controlling for neuroticism, grit, and learning goal orientation, which are all linked to wellbeing.

#### 3.2.5. Exploratory Question: Do Demographics Relate to IMI Scores?

Like Study 1, we also explored whether Iterative Mindsets were related to demographic variables, specifically age, gender, and race. The overall IMI was not significantly related to age, *r* = 0.09, *p* = 0.113, but the practice factor correlated with age, *r* = −0.11, *p* = 0.041, as did iterate (*r* = 0.15, *p* = 0.004) and assess (*r* = 0.11, *p* = 0.038). In line with Study 1, there were no significant differences in IMI by gender (*t*(338.44) = 0.89, *p* = 0.377, *d* = 0.095), and there were no significant differences in practice (*t*(343) = −0.94, *p* = 0.346, *d* = −0.10), iterate (*t*(343) = 0.94, *p* = 0.350, *d* = 0.10), or assess (*t*(341.47) = 1.44, *p* = 0.150, *d* = 0.155) by gender.^2^ We also ran a MANOVA to examine the differences in IMI by race. In line with Study 1, there were no significant differences in IMI or the factors by race (*p* values for univariate tests ranged from 0.617 to 0.984). However, considering the unequal cells and the limited number of participants for some categories, these results should be interpreted with caution.

### 3.3. Study 2 Discussion

We first confirmed the 12-item, three-factor solution, which we called practice, iterate, and assess. We then used the total scale and factors to examine the construct and predictive validity. For example, the Iterative Mindset and factors were correlated positively with grit and learning goals, and only small relations emerged with performance goals. In terms of predictive validity, the overall IMI score and the factors predicted weight-loss success, although the effects were small. Additionally, the IMI is positively related to self-efficacy and wellbeing, with fairly strong correlations. Statistical mediation suggests that the link between Iterative Mindsets and wellbeing can be explained, at least in part, by self-efficacy. This finding is in line with an overall theoretical process model found in past mindset research that outlines how self-efficacy and expectations are key psychological constructs linking mindsets to outcomes (e.g., Burnette et al., 2013, 2023). Finally, the IMI and factors were not related to demographics, except for age.

## 4. Overall Discussion

### 4.1. Summary of Findings

We proposed that Iterative Mindsets can neutralize inevitable challenges and failures to help individuals achieve sustainable behavior change and meaningful personal growth. We focused on three aims: factor structure, construct validity, and predictive utility. We first investigated the Iterative Mindset Inventory (IMI) through exploratory and confirmatory factor analyses and identified a three-factor structure that captured each component Namely, we defined an iterative mindset as the belief that enduring personal transformation is achieved through ongoing strategic adjustments (iterate), deliberate habit building (practice), and a constructive interpretation of failures (assess). In addition to a clear three-factor solution, the assessment had good test–retest reliability and correlated in expected ways with the Big Five personality traits, other mindset measures, and related psychological constructs, such as grit and goal orientations. Furthermore, we assessed the relationship with weight-loss journeys, replicating early work, finding that Iterative Mindsets may help to distinguish between individuals who are successful long-term from those who struggle. However, these effects are small. Additionally, the findings from Study 2 suggest that stronger Iterative Mindsets are positively associated with general self-efficacy and wellbeing.

### 4.2. Theoretical and Practical Applications

Overall, we merged neuroscience evidence with a mindset theoretical perspective, aligning our iterative mindset approach to personal growth within positive psychology with a focus on striving for self-improvement goals, especially those requiring lasting behavior change. We suggested that the Iterative Mindset may be an important tool in the pursuit of personal growth because of its potential to help individuals cope with setbacks to reach goals such as weight loss (Bobinet & Greer, 2023). We also find that the Iterative Mindset, which emphasizes modifications, habit building, and failure management, may be a critical component of overall wellbeing. Although we focused on one self-improvement goal (i.e., weight-loss success) and one aspect of flourishing (wellbeing), we propose that the Iterative Mindset approach can be applied to multiple contexts and outcomes, especially when an orientation towards learning and iteration is needed. Thus, we suggest that future applications could include reducing employee burnout, encouraging resilience, and developing healthy habits, all of which can contribute to improved mental and physical health. We also hope that this initial theoretical and measurement foundational work encourages the development of interventions that foster a stronger Iterative Mindset to improve personal growth.

Additionally, we identified one potential mechanism by which Iterative Mindsets may contribute to wellbeing, which can pave the way for both basic and practical extensions. For example, neutralizing negative thoughts and evaluations by moving away from shame, blame, and the self’s inadequacies and instead towards iteration and practice can help individuals to preserve their positive view of themselves and their abilities, rather than viewing failure as a signal of one’s lack of worth. In doing so, individuals may be able to recover more quickly from setbacks and continue on their path of self-improvement. This is particularly evident in findings from Study 2, which reveal strong positive relationships between Iterative Mindsets, their factors, and self-efficacy. Yet, self-efficacy is only one of many potential mechanisms. Indeed, we speculate that an Iterative Mindset can neutralize failure by not activating the motivation kill switch, namely the habenula, which acts as a central hub for a variety of psychological outcomes, including behavioral regulation (Hikosaka, 2010). We hope these initial findings pave the way for future work empirically examining the neuroscientific underpinnings of the link between Iterative Mindsets and personal growth.

### 4.3. Limitations and Future Directions

Furthermore, there are limitations to note that can serve as a springboard for future inquiry. For example, although we conducted statistical tests of mediation, due to the correlational nature of this work, these analyses cannot provide causal evidence (Pirlott & MacKinnon, 2016). Furthermore, we identified only one mediator, although there are likely multiple pathways. For example, individuals with stronger growth mindsets show greater activation of areas of the brain that are connected to monitoring and adjusting errors, suggesting they may be more open to feedback about their mistakes (Moser et al., 2011; Myers et al., 2016). In addition to examining the underlying neuroscientific-driven mediations, additional research, especially that implementing interventions, can draw on the mindset intervention effectiveness (MIE) model, which outlines multiple processes linking mindsets to distal outcomes, such as goal achievement and wellbeing (Burnette et al., 2023). For example, one potential serial mediation model building on the MIE might be to examine if Iterative Mindsets work by neutralizing negative emotions in the face of setbacks, which thereby triggers more active behavioral coping, such as iteration and practice. This work is especially relevant considering the small variance explained in the models predicting the distal weight-loss success outcome. Yet, Iterative Mindsets showed a strong correlation with general self-efficacy. These findings are in line with the MIE model, which outlines larger impacts of growth mindset on more proximal outcomes such as self-efficacy but much smaller effects on distal outcomes such as performance (Burnette et al., 2023). Overall, more work is needed that identifies additional theoretically driven mechanisms of change and incorporates causal tests of mediation (e.g., Pirlott & MacKinnon, 2016).

Furthermore, methodological limitations that can be addressed in future work include replicating and extending the findings with confirmatory approaches and pre-registered replications to bolster evidence. Additionally, future work could incorporate parallel analysis to replicate findings and explore the ideal number of factors and factor structure, along with addressing problems related to overfitting (Horn, 1965). Furthermore, the current studies were conducted with primarily WEIRD samples (Henrich et al., 2010), with all of the participants residing in the United States and over three-quarters of the sample identifying as White. The limited diversity raises important questions for future research. For example, how might cultural norms and values shape mindsets? Considering cultural differences in approaches to self-improvement (Tsai et al., 2016) and learning vs. performance (Dekker & Fischer, 2008), Iterative Mindsets may be easier to foster in cultures that provide more support for growth (Walton & Yeager, 2020). Additionally, in cultures that emphasize individual agency (e.g., many Western contexts), participants may report stronger Iterative Mindsets, whereas in cultures that emphasize conformity (e.g., some East Asian contexts), participants may be discouraged from adopting beliefs and behaviors that embrace failure (e.g., Kim & Markus, 1999). Understanding these cultural dynamics can help refine theories of iteration and ensure that interventions are appropriately tailored across diverse populations.

Additionally, more work is needed to address the smaller effect sizes and determine the conditions under which the Iterative Mindset might facilitate self-improvement. For example, future work should identify whether domain-specific Iterative Mindsets of weight are stronger correlates of weight-loss journeys, as prior work in other areas, such as computer programming and stress, illustrates how domain-specific mindsets (e.g., a mindset about computer programming; general vs. specific stress mindset measures) are better predictors of outcomes in that domain (e.g., Crum et al., 2013; Scott & Ghinea, 2014). Additionally, we used a single-item assessment of weight-loss success—one that was self-reported. However, weight loss is complex, and self-reports likely fail to capture important behavioral change strategies and details. Thus, additional work is needed that includes assessments of the automaticity of habits, specific behavior changes, and indicators of improved health. Finally, personal growth encompasses more than being efficacious, reaching goals, and feeling good. It also encompasses productivity, improved physical health, learning from setbacks, and more. We hope that this initial exploration of links between Iterative Mindsets, personal growth, and wellbeing paves the way for inquiry into a varied set of outcomes.

### 4.4. Conclusions

We suggest that the Iterative Mindset offers a promising framework for understanding sustained personal growth, with an emphasis on self-improvement. Findings from the two studies demonstrate the Iterative Mindset Inventory’s psychometric properties and its potential to predict positive outcomes, particularly in contexts like weight-loss journeys and wellbeing. We sought to integrate neuroscience findings with mindset research to strengthen both theory and application by grounding personal growth constructs in biological systems. Future research should test the proposed mechanism of the lateral habenula as a critical neuroanatomic correlate and could also explore the generalizability of the approach across various aspects of personal growth. We hope that this initial foundational theoretical and measurement work can be used to develop interventions that leverage the core principles of an Iterative Mindset to address a host of personal growth outcomes. Furthermore, building on past approaches to growth mindset interventions (e.g., Burnette et al., 2023), future work should seek to incorporate overall process models that outline why, how, when, and for whom Iterative Mindset interventions may work best to improve personal growth. We hope this early theorizing paves the way for such future explorations.

## Figures and Tables

**Table 1:** Factor loadings for final 12-item measure.

Item	Factor 1: Practice	Factor 2: Iterate	Factor 3: Assess
1. I know that constant practice is the only way to make changes.	0.779		
2. It is critical to practice my routines.	0.784		
3. Good habits take practice.	0.806		
4. I find it valuable to repeat things until they become a habit.	0.851		
5. I can adapt as needed to help me reach my goals.		0.689	
6. I’m willing to try new things, even if there’s a chance it won’t work.		0.677	
7. I believe I should try to change my approach first before giving up.		0.617	
8. Even if progress is slow, I can find new strategies.		0.781	
9. I do not dwell on or think much about past mistakes.			0.811
10. When I struggle to change my behavior, I don’t blame myself.			0.695
11. I avoid feeling shame after setbacks.			0.749
12. If a goal isn’t working for me, I don’t blame myself.			0.823
% Variance Explained	39.470	11.934	4.577

**Table 2:** Study 1. Key correlations between variables.

	*M*	*SD*	1	2	3	4	5	6	7	8	9	10	11
1. IMI	5.13	0.77	--	--	--	--	--	--	--	--	--	--	--
2. Practice	5.70	0.82	0.64 ***	--	--	--	--	--	--	--	--	--	--
3. Iterate	5.63	0.80	0.80 ***	0.54 ***	--	--	--	--	--	--	--	--	--
4. Assess	4.05	1.40	0.81 ***	0.15 ***	0.42 ***	--	--	--	--	--	--	--	--
5. Mindsets of Intelligence	4.51	1.75	0.05	0.09 **	0.13 ***	−0.04	--	--	--	--	--	--	--
6. Mindsets of People	4.55	1.41	0.18 ***	0.18 ***	0.23 ***	0.07	0.68 ***	--	--	--	--	--	--
7. Extraversion	2.63	1.19	0.27 ***	0.07	0.29 ***	0.24 ***	0.07 *	0.12 ***	--	--	--	--	--
8. Agreeableness	3.43	1.05	0.32 ***	0.06	0.21 ***	0.37 ***	0.14 ***	0.23 ***	0.25 ***	--	--	--	--
9. Conscientiousness	4.01	0.91	0.36 ***	0.15 ***	0.34 ***	0.30 ***	0.02	0.06	0.27 ***	0.24 ***	--	--	--
10. Neuroticism	2.69	1.21	−0.43 ***	−0.08 *	−0.34	−0.47 ***	−0.04	−0.11 ***	−0.34 ***	−0.36 ***	−0.41 ***	--	--
11. Openness	3.83	0.95	0.16 ***	0.11 ***	0.22 ***	0.06	0.15 ***	0.16 ***	0.15 ***	0.02	0.10 **	−0.10 **	--

***. Correlation is significant at the 0.001 level (2-tailed). **. Correlation is significant at the 0.01 level (2-tailed). *. Correlation is significant at the 0.05 level (2-tailed).

**Table 3:** Study 2, key correlations between variables.

	*M*	*SD*	1	2	3	4	5	6	7	8	9	10
1. IMI	5.06	0.80	--	--	--	--	--	--	--	--	--	--
2. Practice	5.72	0.89	0.55 ***	--	--	--	--	--	--	--	--	--
3. Iterate	5.60	0.91	0.80 ***	0.38 ***	--	--	--	--	--	--	--	--
4. Assess	3.86	1.46	0.80 ***	0.05	0.46 ***	--	--	--	--	--	--	--
5. Neuroticism	2.46	1.03	−0.60 ***	−0.07	−0.54 ***	−0.60 ***	--	--	--	--	--	--
6. Grit	3.49	0.77	0.52 ***	0.13 *	0.49 ***	0.46 ***	−0.60 ***	--	--	--	--	--
7. Learning Goal Orientation	5.35	1.04	0.56 ***	0.34 ***	0.65 ***	0.30 ***	−0.36 ***	0.43 ***	--	--	--	--
8. Performance Goal Orientation	5.43	0.92	−0.04	0.21 ***	−0.01	−0.18 ***	0.17 ***	−0.21 ***	0.03	--	--	--
9. General Self-Efficacy	31.46	5.34	0.64 ***	0.23 ***	0.68 ***	0.48 ***	−0.65 ***	0.56 ***	0.51 ***	−0.02	--	--
10. Wellbeing	3.70	0.74	0.68 ***	0.24 ***	0.61 ***	0.58 ***	−0.77 ***	0.66 ***	0.49 ***	−0.07	0.70 ***	--

***. Correlation is significant at the 0.001 level (2-tailed). *. Correlation is significant at the 0.05 level (2-tailed).

## Data Availability

Data is available upon reasonable request.

## Notes

1 We had also measured Trait Hope but do not include it in the manuscript, as initial analyses revealed it was highly correlated with all the constructs, including IMI (r = 0.71, *p* < 0.001), learning goal orientation (r = 0.68, *p* < 0.001), neuroticism (r = −0.61, *p* < 0.001), and grit (r = 0.55, *p* < 0.001), suggesting it may not actually be measuring trait hope and may be capturing a number of different related theoretical ideas.

2 For the overall IMI and assess, we violated homogeneity of variance according to Levene’s test and, therefore, included the results when equal variances are not assumed (Fein et al., 2022).
